# Nigerian parents’ knowledge of COVID-19 and effect of lockdown on monthly earnings: an online survey

**DOI:** 10.11604/pamj.supp.2020.37.1.25335

**Published:** 2020-12-30

**Authors:** Datonye Christopher Briggs, Kattey Amos Kattey

**Affiliations:** 1 Department of Paediatrics, Rivers State University Teaching Hospital, Port Harcourt, Rivers State, Nigeria,; 2 Department of Community Medicine and Public Health, Rivers State University Teaching Hospital, Port Harcourt, Nigeria

**Keywords:** Nigeria, knowledge, income, COVID-19, pandemic, SARS-CoV-2, virus, parents

## Abstract

**Introduction:**

the COVID-19 pandemic has devastated every sector leading to untold hardship, unplanned loss of jobs and drastic reductions in the income of families across the world. This survey assessed the knowledge of COVID-19 among Nigerian parents and its effect on their monthly income.

**Methods:**

an online cross-sectional survey was conducted from May 9 - June 8, 2020, among parents/guardians with children/wards in the paediatric age-range during the lockdown stage of the outbreak in Nigeria. Snowball sampling technique was used to recruit 260 respondents. The questionnaire was administered on a Google doc form and distributed via the internet. Chi-square was used to test for differences, and statistical significance was set at p-value less than 0.05 and a 95% confidence level.

**Results:**

the mean age was 39.6 years (SD = 7.3), comprising 105 (40.4%) males, 239 (91.9%) married, and 167 (64.2%) with tertiary education. Only 29 (11.2%) had good knowledge of COVID-19. Interestingly, low-income earners were more likely to have good knowledge of COVID-19 than middle/high-income earners. The monthly incomes of 191 (73.5%) respondents were affected. Females, those with secondary education and below, and low-income earners were more likely to have their incomes affected.

**Conclusion:**

parents/guardians have poor knowledge of COVID-19. Also, the monthly income of parents/guardians have been affected by the lockdown measures; most affected were females, those with a secondary level of education and below and low-income earners. Their poor knowledge and the impact on their income may further impair their preparedness to combat the spread of COVID-19.

## Introduction

Parents play a key role as essential front-line workers in curbing the spread of the ongoing coronavirus pandemic [1]. The onus rests on parents to take responsibility and be fully aware of the novel COVID-19 disease that has significantly affected not only their families but has also taken a toll on their sources of income and without warning, brought drastic changes to work-life and halted in many cases, what was considered normal social interaction. Parents should, therefore, be knowledgeable and understand what is and how the coronavirus outbreak can be spread and prevented [2] since part of their duty is to effectively teach and empower their children to safeguard themselves from the virus, which in turn would help limit spread by ensuring preventive measures are strictly adhered to. The Nigerian Center for Disease Control (NCDC), Federal and State Ministries of Health, social media communication houses, public and private-owned health institutions have embarked on elaborate media campaigns to enlighten the Nigerian populace on the novel virus, its modes of transmission and preventive measures [3]. Up-to-date information is also made available including parenting tips on the NCDC website as a helpful resource for parents in Nigeria [4]. Furthermore, with the restriction of movement, COVID-19 has altered household income by affecting parents´ sources of livelihood and may force poor families to cut back on essential food and healthcare expenditures [5,6]. This online survey, therefore, aimed to explore Nigerian parents´ knowledge of COVID-19 and assess the effect of the lockdown on their monthly earnings.

## Methods

Nigeria has 36 states including the Federal Capital Territory (FCT) Comprising of six geopolitical viz: south-east {Abia, Anambra, Ebonyi, Enugu, Imo}, south-south {Akwa-Ibom, Bayelsa, Cross River, Rivers, Delta, Edo}, south-west {Ekiti, Lagos, Ogun, Ondo, Osun, Oyo}, north-east {Adamawa, Bauchi, Borno, Gombe, Taraba, Yobe}, north-central {Benue, Kogi, Kwara, Nasarawa, Niger, Plateau and FCT}, and north-west {Jigawa, Kaduna, Kano, Katsina, Kebbi, Sokoto, Zamfara}. This was used to ascertain the spread of the respondents and classify respondents accordingly. This study was a cross-sectional survey, conducted over one month among consenting parents or caregivers who had children within the paediatric age group (0-18 years).

**Inclusion criteria:** consenting parents/guardians who resided in any state in Nigeria affected by COVID-19 outbreak and had experienced either partial or total lockdowns, owned a smartphone with access to the internet and had the WhatsApp application installed.

**Exclusion criteria:** respondents (either single or married) who did not have children/wards within the paediatric age group were excluded. The sample size was estimated using the Cochran formula for proportions:

$${\text{N}}_{\text{0}}={\text{Z}}^{\text{2}}{\text{pq/e}}^{\text{2}}$$

An assumption of p = 62.7% was done, obtained from the prevalence of “good” knowledge towards coronavirus disease outbreak from a previous study [7]. N_0_= sample size, Z = 1.96 and e = 6%, hence, N_0_= 250. Using a non-probability (snowball) sampling technique, a total of 260 respondents were recruited through the sharing of the online survey link. The link was shared on several WhatsApp groups with the instruction that recipients could share with others. A questionnaire was used which was administered through a Google Doc form and widely distributed through an instant message via “WhatsApp Application”. The semi-structured questionnaire was used to derive information about the respondents´ sociodemographics and questions which assessed their knowledge of COVID-19 as follows: the respondents´ knowledge of the correct name of the virus using four options of which only one was correct- coronavirus, COVID-19 virus, SARS-CoV-2 and SARS-CoV-1; respondent´s knowledge of the modes of transmission using five options (with multiple correct answers)- droplets in a cough, contact with bodily secretions, contact with contaminated surfaces, airborne and eating infected food/drinks. For the knowledge of the preventive measures taken to curb the spread, respondents were assessed using a 6-point checklist (with multiple correct answers) developed from the WHO guidelines [8] which comprised of wearing face or surgical mask, social distancing, regular washing of hands with soap, use of hand sanitizers for soiled hands, coughing into bare hands and wearing hand gloves. The three categories of questions had a total of 15 options out of which were 8 correct answers. Each correct answer was given a score of 2. A wrong answer led to subtraction of 1. The highest possible score was 16. Based on the total knowledge score, respondents were categorized as having: good knowledge = 13-16 points, intermediate knowledge = 10 - 12 points and low knowledge = 9 points and below. Questions on whether the restrictions of movement affected monthly income earnings and to what extent, was also inquired. Respondents average monthly income earnings were categorized into three groups, adapted from a survey by renaissance capital [9]: less than N100, 000 = low income; N100,000 - N499,999 = middle income; and N500,000 and above as high-income earners.

**Data analyses:** completed questionnaires were automatically imported to excel spreadsheet and analysed using SPSS v 26 (SPSS Inc, Chicago, Illinois, USA). Simple frequencies and cross tables were performed and relevant tables were developed. The associations between the level of knowledge and socio-demographic characteristics of respondents were compared using the chi-square test and p-value of less than 0.05 was considered statistically significant. For the logistic regression part of the analysis, all the categorical variables were made dichotomous. For instance, level of knowledge which has three (3) values of good knowledge, moderate knowledge and poor knowledge, was re-arranged thus for logistic regression: good/moderate knowledge vs poor knowledge. The income level of respondents (low income, middle income, and high income) was categorized into low income and middle/high income for logistic regression.

**Ethical considerations:** all respondents were pre-informed about the research aims and assured of confidentiality. Respondents were also informed that by choosing to access the survey, they were consenting to participate voluntarily. Their responses were anonymous and the researchers did not know the identity of all the respondents.

## Results

A total of 272 respondents filled the online survey but 12 were excluded.

**Demographic characteristics of respondents:** of the 260 respondents included in the study, there were 105 males (40.4%), the mean age of the respondents was 39.6 years (SD = 7.3), 239 (91.9%) were married, 167 (64.2%) had tertiary education, 131 (50.4%) had three to four children and 83 (31.9) of the respondents, earned less than N100,000.00 monthly (Table 1). All respondents were aware of COVID-19 pandemic and were residing in states affected by either partial or total lockdown. Although respondents from all six geopolitical zones in Nigeria responded to the survey, only five zones were included in the analyses; one hundred and seventy-one (65.8%) of the respondents were from the south-south, (27, 10.4%) were from north-central and the least represented regions were the north-west (9, 3.5%) and north-east (0, 0%) geo-political zones respectively (Table 1).

**Table 1:** socio-demographic characteristics of respondents

Variables	Frequency	Percent (%)
**Gender**		
Female	155	59.6
Male	105	40.4
**Marital status**		
Married	239	91.9
Single	10	3.8
Separated	9	3.5
Divorced	2	0.8
**Educational status**		
Tertiary	167	64.2
Secondary and below	93	35.8
**Number of children under respondents' care**		
1-2	112	43.1
3-4	131	50.4
More than 4	17	6.5
Age categories of respondents' child(ren)^**^		
0-4 years	156	60.0
5-9 years	150	57.7
10-14 years	90	34.6
15-18 years	43	16.5
**Monthly income**		
Less than N100,000	83	31.9
N100,000 - N199,999	52	20.0
N200,000 - N299,999	46	17.7
N300,000 - N399,999	27	10.4
N400,000 - N499,999	16	6.2
Above N500,000	36	13.8
**Geopolitical zones of respondents**		
South-south	171	65.8
South-east	18	6.9
South-west	35	13.5
North-central	27	10.4
North-west	9	3.5
North-east	0	0.0

** Multiple responses apply

**Knowledge of respondents about COVID-19 - causative agent, routes of transmission and preventive measures:** only 53 (20.4%) of the respondents knew the correct name of the virus causing COVID-19 to be SARS-CoV-2. With regards to the possible routes of transmission, 42 (16.2%) could correctly pick the correct routes of transmission of the virus. Regarding the preventive measures highlighted, only 9 (3.5%) correctly identified the correct combination of measures recommended to curb the spread of the virus.

**The overall level knowledge of respondents:** generally, 29 (11.2%) of respondents had “good” knowledge about the COVID-19 outbreak, 115 (45.2%) had “moderate” knowledge and 116 (44.6%) had “poor” knowledge.

**Effect on monthly earnings:** a total of 191 (73.5%) of the respondents stated that their monthly income was affected by the lockdown measure imposed to help enforce physical distancing and spread of the virus.

**Bivariate analysis:** on bivariate analysis, the educational status and level of income the respondents were associated with their level of knowledge of COVID-19 (Table 2). The sex of respondents, educational status, and income level was associated with the affectation of their income (Table 3).

**Table 2:** relationship between the level of knowledge of COVID-19 and socio-demographic characteristics

Variables	Good knowledge (%)	Moderate knowledge (%)	Poor knowledge (%)	Chi-square	P-value
**Educational status**					
Tertiary	26 (15.6)	71 (42.5)	70 (41.9)		
Secondary and below	3 (3.2)	44 (47.3)	46 (49.5)	9.23	0.01^*^
**Sex of respondent**					
Female	17 (11.0)	74 (47.7)	64 (41.3)		
Male	12 (11.4)	41 (39.0)	52 (49.5)	2.03	0.36
**Number of children in respondent’s care**					
1-2	19 (17.0)	46 (41.1)	47 (42.0)		
3-4	9 (6.9)	63 (48.1)	59 (45.0)		
More than 4	1 (5.9)	6 (35.3)	10 (58.8)	7.92	0.10
**Level of income**					
High income	7 (19.4)	17 (47.2)	12 (33.3)		
Middle income	18 (12.8)	65 (46.1)	58 (41.1)		
Low income	4 (4.8)	33 (39.8)	46 (55.4)	9.68	0.046^*^
**Access to internet services at home**					
Yes	29 (11.9)	108 (44.4)	106 (43.6)		
No	0 (0.0)	7 (41.2)	10 (58.8)	2.89	0.24

* Statistically significant (p<0.05)

**Table 3:** socio-demographic characteristics of respondents who report income being affected by COVID-19 lockdown measure

Variables	Income affected (%)	Income not affected (%)	Chi-square	P-value
**Gender**				
Females	104 (67.1)	51 (32.9)		
Males	87 (82.9)	18 (17.1)	7.98	0.01^*^
**Marital status**				
Married	173 (72.4)	66 (27.6)		
Single	8 (80.0)	2 (20.0)		
Separated	9 (100.0)	0 (0.0)		
Divorced	1 (50.0)	1 (50.0)	4.43 (Fisher's exact)	0.21
**Educational status**				
Tertiary	111 (66.5)	56 (33.5)		
Secondary and below	80 (86.0)	13 (14.0)	11.72	0.001^*^
**Number of children under respondents care**				
1-2	82 (73.2)	30 (26.8)		
3-4	96 (73.3)	35 (26.7)		
More than 4	13 (76.5)	4 (23.5)	0.09	0.96
**Income level**				
High income	25 (69.4)	11 (30.6)		
Middle income	92 (65.2)	49 (34.8)		
Low income	74 (89.2)	9 (10.8)	15.66	< 0.001^*^

* Statistically significant (p<0.05)

**Multiple regression analysis:** after controlling for educational status, respondents with a low level of income were nearly twice likely to have good knowledge of COVID-19 causation, transmission and prevention than those with middle or high income (Adjusted OR: 1.83, 95% CI= 1.05-3.16) (Table 4). In addition, after controlling for the influence of other variables, females were 4 times more likely to report that their incomes were affected than males (Adjusted OR= 4.45; 95% CI= 1.20-9.92) (Table 4). Respondents with a secondary level of education and below were about 3 times more likely to have their income affected than those with a tertiary level of education (Adjusted OR= 2.65; 95% CI= 1.30-5.40), while the parents/guardians with low-income were thrice likely to have their income affected than those with middle or high income (Adjusted OR= 3.40; 95% CI= 1.78-6.53) (Table 4).

**Table 4:** multiple regression analysis of factors associated with respondents whose income was affected

	Crude		Adjusted	
Variables	OR (95% CI)	P-value	OR (95% CI)	P-value
**Sex**				
Female	2.37(1.29 - 4.35)	0.005^*^	4.45 (1.20 - 9.92)	<0.001^*^
Male				
**Educational status**				
Secondary and below	3.11 (1.59 - 6.06)	0.001^*^	2.65 (1.30 - 5.40)	0.007^*^
Tertiary				
**Income level**				
Low	4.22(1.98 - 9.00)	<0.001^*^	3.40 (1.78 - 6.53)	<0.001^*^
Middle/High				

* Statistically significant (p<0.05)

## Discussion

The study set out to assess Nigerian parents´ knowledge of the COVID-19 pandemic and also explore its effect on their monthly income. The overall knowledge of respondents about the ongoing COVID-19 pandemic was unexpectedly poor as only one-tenth of respondents had “good knowledge”. This was quite shocking to observe even though the pandemic had been ongoing for three months before the study with widespread social media, government campaign and already on-going lockdown as mitigating measures. Our finding was similar to a study in Cameroon [10] where only 21.9% of respondents had good knowledge of COVID-19 and the authors attributed their findings to be due to the relatively early timing of their study when only a few cases had been reported and also the likely adjusting to accommodate the on-going pandemic superimposed on an already on-going crisis. Our study, however, compares unfavourably to a study in China [11] where it was found that respondents had a high overall correct rate of 90% on the knowledge of COVID-19 despite being in the very early stage of the epidemic and was likely because respondents were from an elite and learned cohort. Although the sample characteristics of respondents in the China study [11] were similar to this present study, the dissimilar findings may demonstrate the initial disbelief, and perhaps indifference towards the reality of the COVID-19 pandemic, despite the various channels of information employed by the government to disseminate health information about the novel coronavirus.

A study by Olapegba *et al*. [12] that comprised of 1357 respondents in Nigeria, aged between 15 - 70 years reported that respondents had a relatively high knowledge of COVID-19. The study also found that 94.25% of them correctly identified regular handwashing and social distancing as a way of preventing infection. It was hence, worrisome that only a fifth of the respondents in our study could correctly identify the specific name of the virus as SARS-CoV-2, which causes COVID-19 disease, only 16% of respondents could correctly identify the correct modes of transmission of the virus and only 3.5% were able to identify the correct measures of preventing the spread of the virus, despite having similar sociodemographic characteristics. Having heard about the 'coronavirus' is quite different from knowing exactly what it entails and knowing only one measure to curb the spread is insufficient. Our study identified a gap in in-depth knowledge which has dire implications and could suggest the possibility of rapid community spread of the virus since knowledge is incomplete and taking into cognizance the usual communal/co-housing style of living as extended family households being predominant in our setting. Therefore, the likelihood of person-to-person spread would be unsurprisingly high with a consequent decrease in the possibility for contact tracing of contacts with confirmed COVID-19 positive cases.

Nonetheless, there was a significant relationship between the level of education and income strata with the level of knowledge of the respondents. This relationship between the knowledge of COVID-19 and the respondents´ level of education may be because the more educated people are, the more likely they are to be properly informed. This was similarly reported in the other studies in Ghana and China [7,11]. In contrast to what might be expected theoretically, our study revealed that after controlling for the educational status of our respondents, those with low income were about twice likely to have good knowledge compared to respondents of middle/high-income class. We can only hypothesize that the low-income earners are more likely to pay attention to information shared over the TV/ radio because they may not be able to afford healthcare services out-of-pocket knowing the predicament the lockdown measures had forcefully imposed on their daily wages.

The obvious lack of knowledge of the modes of transmission and preventive measures to curb the spread of the virus among respondents in this study could plausibly affect their attitude and invariably safe practice of these measures. More importantly, it may affect how they communicate with their children. Several studies have linked good knowledge of COVID-19 to high population compliance and attitudes to the implementation of government measures [11,13]. Our finding may not be unrelated to the continued lack of compliance to the NCDC guidelines with the inappropriate wearing of face masks and/or face shields, as well as disregard for physical distancing rules which has been similarly reported in another Nigerian study by Lawani-Luwaji *et al*. [14] or the initial disbelief in the fact that the virus was a political hoax since the death toll was not alarming as was initially envisaged.

The majority of respondents cited their income being negatively affected by the lockdown measure instituted to curb the spread of the virus. This has been similarly reported and envisaged [15]. The COVID-19 had led to reduced economic activities bringing about forced inflation in staple food items due to disruption in daily income and a sudden rise in unemployment and poverty [6]. Our study demonstrated that females, parents/guardians with a secondary or primary level of education, and respondents of the low-income group had their incomes significantly affected by the on-going pandemic. This highlights the fact that the impoverished are the worst hit by the on-going pandemic and the mitigating measures because the resulting hunger, unemployment and poverty are inevitably linked to the pandemic [16]. In this study, females were significantly more likely to have their income affected, after controlling for other variables. This might be due to the financial dependence of females on their male partners; thus, when the income of their male partners is affected, the females would, in turn, have a further worsening of their income. The sudden inability for parents to support their families due to the pandemic will work against curbing the spread of the virus because there would need to move about in search for food, a job and physical contact.

The findings in this study identified a knowledge gap about COVID-19 and buttress the need for continued community health education and promotion with proper risk communication to be done at all levels. It is useful for both public health policy-makers and healthcare workers to recognize target populations for COVID-19 prevention and health education. The strength of the study lies in the fact that respondents were recruited during a critical period during the early stage of the COVID-19 pandemic and lockdown in Nigeria and not merely retrospective limiting the likelihood of bias. However, the sample size is small with limited sample representativeness and lack of assessment of parents´ attitudes and practices towards COVID-19 was not done. The findings are limited to parents who reside in Nigeria with considerable knowledge in the use of the internet and the ability to read and understand in the English Language. More so, the majority of respondents had a tertiary level of education. Vulnerable populations and illiterates were not captured in the study so generalization of findings should be with caution.

## Conclusion

Parents have poor knowledge of COVID-19 and their monthly income earnings are considerably affected by the lockdown, which may impair their preparedness to combat the spread of the virus due to the effect of COVID-19.

### What is known about this topic

- COVID-19 has significantly impacted on families the world over;
- Knowledge of COVID-19 is rapidly changing and influences the attitudes and practice of mitigating measures;
- Little is known about Nigerian parents' knowledge of COVID-19 and the perceived effect of the lockdown on their monthly income.

### What this study adds

- This study confirms a considerable lack of knowledge of COVID-19 among Nigerian parents and highlights a negative effect on their income during the lockdown;
- The findings in this study buttress the need for continued community health education and promotion with proper risk communication to be done at all levels and are useful for both public health policy-makers and healthcare workers to recognize target populations for COVID-19 prevention and health education.
